# Galcanezumab for the prevention of high frequency episodic and chronic migraine in real life in Italy: a multicenter prospective cohort study (the GARLIT study)

**DOI:** 10.1186/s10194-021-01247-1

**Published:** 2021-05-03

**Authors:** Fabrizio Vernieri, Claudia Altamura, Nicoletta Brunelli, Carmelina Maria Costa, Cinzia Aurilia, Gabriella Egeo, Luisa Fofi, Valentina Favoni, Giulia Pierangeli, Carlo Lovati, Marco Aguggia, Florindo d’Onofrio, Alberto Doretti, Paola Di Fiore, Cinzia Finocchi, Renata Rao, Francesco Bono, Angelo Ranieri, Maria Albanese, Sabina Cevoli, Piero Barbanti, for the GARLIT Study Group, Chiara Capogrosso, Davide Bertuzzo, Daniele Spitaleri, Stefano Messina, Francesca Trogu, Fabio Frediani, Ottavia Baldi, Francesca Schiano Di Cola, Lucia Manzo, Angelo Pascarella, Gennaro Alfieri

**Affiliations:** 1 Headache and Neurosonology Unit, Campus Bio-Medico University Hospital, Via Alvaro del Portillo, 200, 00128 Rome, Italy; 2 Headache and Pain Unit, IRCCS San Raffaele Pisana, Rome, Italy; 3 IRCCS Istituto delle Scienze Neurologiche di Bologna, Bologna, Italy; 4 Headache Center, Neurology Unit, University Hospital L. Sacco, Milan, Italy; 5 Neurology and Stroke Unit, Asti Hospital, Asti, Italy; 6 Neurology Unit, San Giuseppe Moscati Hospital, Avellino, Italy; 7 Department of Neurology, Stroke Unit and Laboratory of Neuroscience, Istituto Auxologico Italiano, IRCCS, Milan, Italy; 8 Headache Center, Neurology and Stroke Unit, S. Carlo Borromeo Hospital, Milan, Italy; 9 IRCCS Ospedale Policlinico San Martino, Genoa, Italy; 10 Neurology Unit, Department of Clinical and Experimental Sciences, University of Brescia, Brescia, Italy; 11 Center for Headache and Intracranial Pressure Disorders, Neurology Unit, A.O.U. Mater Domini, Catanzaro, Italy; 12 Headache Centre, Neurology and Stroke Unit, A. Cardarelli Hospital, Naples, Italy; 13 Headache Center, Neurology Unit, Tor Vergata University Hospital, Rome, Italy; 14 Department of Systems Medicine, Tor Vergata University, Rome, Italy; 15 San Raffaele University, Rome, Italy

**Keywords:** Calcitonin gene-related peptide, Monoclonal antibodies, Migraine treatment, Real world

## Abstract

**Background:**

The clinical benefit of galcanezumab, demonstrated in randomized clinical trials (RCTs), remains to be quantified in real life. This study aimed at evaluating the effectiveness, safety and tolerability of galcanezumab in the prevention of high-frequency episodic migraine (HFEM) and chronic migraine (CM) in a real-life setting.

**Methods:**

This multicenter prospective observational cohort study was conducted between November 2019 and January 2021 at 13 Italian headache centers. Consecutive adult HFEM and CM patients clinically eligible were enrolled and treated with galcanezumab subcutaneous injection 120 mg monthly with the first loading dose of 240 mg. The primary endpoint was the change in monthly migraine days (MMDs) in HFEM and monthly headache days (MHDs) in CM patients after 6 months of therapy (V6). Secondary endpoints were the Numerical Rating Scale (NRS), monthly painkiller intake (MPI), HIT-6 and MIDAS scores changes, ≥50% responder rates (RR), the conversion rate from CM to episodic migraine (EM) and Medication Overuse (MO) discontinuation.

**Results:**

One hundred sixty-three patients (80.5% female, 47.1 ± 11.7 years, 79.8% CM) were included. At V6, MMDs reduced by 8 days in HFEM and MHDs by 13 days in CM patients (both *p* < .001). NRS, MPI, HIT-6 and MIDAS scores significantly decreased (*p* < .001). Ten patients (6.1%) dropped out for inefficacy and classified as non-responders. Patients with ≥50%RRs, i.e. responders, were 76.5% in the HFEM and 63.5% in the CM group at V6. Among CM patients, the V6 responders presented a lower body mass index (*p* = .018) and had failed a lower number of preventive treatments (*p* = .013) than non-responders. At V6, 77.2% of CM patients converted to EM, and 82.0% ceased MO. Adverse events, none serious, were reported in up to 10.3% of patients during evaluation times.

**Conclusions:**

Galcanezumab in real life was safe, well tolerated and seemed more effective than in RCTs. Normal weight and a low number of failed preventives were positively associated with galcanezumab effectiveness in CM patients.

**Trial registration:**

ClinicalTrials.govNCT04803513.

## Background

Migraine is a very disabling neurological disorder [1], mainly when attacks occur frequently and severely. In these cases, the prescription of preventive drugs is strongly recommended [2]. Most prophylactic medications advised by international guidelines [3, 4] were not specifically developed for migraine pathophysiology. Although beta-blockers, antidepressants and antiepileptics remain worldwide the first-line classes of preventive drugs suggested for both chronic (CM) and episodic (EM) migraine, clinical trials demonstrated their efficacy mostly in EM [4], while topiramate and onabotulinumtoxinA are the only drugs with evidence based on clinical trials in CM [5, 6]. Moreover, adherence to long term oral migraine preventive medications is poor because of adverse events and often inadequate effectiveness [7].

A new era in migraine therapy has recently started with discovering the trigeminal sensory calcitonin gene-related peptide (CGRP) and its role in activating the trigeminovascular pain pathway [8]. Randomized controlled trials (RCTs) have now demonstrated that the specifically designed monoclonal antibodies (mAbs) anti-CGRP receptor, i.e. erenumab [9, 10], and anti the CGRP ligand, i.e. galcanezumab [11–13], fremanezumab [14, 15], and eptinezumab [16] are effective and safe in the prevention of EM and CM. Real-life studies with erenumab have confirmed trials’ findings also in clinical practice [17].

The efficacy and safety of galcanezumab have been established in 3 Phase III RCTs – EVOLVE-1 [11] and EVOLVE-2 [12] in EM and REGAIN [13] in CM patients [18].

Galcanezumab was approved by international drug agencies in 2019 and has been available in Italy for the preventive treatment of high-frequency episodic migraine (HFEM) and CM since November 2019. We reported the first multicentric 3-month follow-up observation, describing the high effectiveness and tolerability of galcanezumab in HFEM and CM patients also in real life [19].

The present observational, multicenter study aims to investigate in real life the effectiveness, safety, and tolerability of galcanezumab in CM and HFEM patients after 6 months of treatment (the GARLIT study).

## Methods

GARLIT is an independent, multicenter, prospective, cohort, real-life study ongoing at 13 Italian headache centers across seven regions from November 2019, with the latest data survey on January 31, 2021.

All consecutive patients aged 18 or older with a diagnosis of HFEM (8–14 migraine days per month) or CM (1.3 ICHD-3) [20], not previously involved in any CGRP mAbs trial, with indication to galcanezumab treatment according to eligibility criteria [21, 22] were considered for enrolment.

Patients were assessed at baseline by a headache expert neurologist with a face-to-face interview using a semi-structured questionnaire addressing socio-demographic factors, clinical migraine features, previous and current acute and preventive migraine treatments, comorbidities and concomitant medications.

Migraine-related dopaminergic and unilateral cranial autonomic symptoms [17], temporal artery turgidity/hyperpulsatility, and allodynia during or between attacks were also investigated [23]. Cranial autonomic symptoms were defined at least one symptom among ipsilateral conjunctival injection, lacrimation, nasal congestion, rhinorrhoea, forehead and facial sweating, miosis, ptosis and/or eyelid oedema. Dopaminergic symptoms were at least one symptom among yawning, somnolence, severe nausea (i.e. requiring specific treatment) and vomiting during prodromes, headache stage or postdromes. Patients were also requested to rate the overall efficacy of triptans in most attacks as none/poor or fair/excellent.

Enrolled patients were requested to carefully fill out a daily headache diary during a run-in month period (baseline) and the entire duration of the study, to report monthly migraine days (MMDs) for HFEM patients, all monthly headache days (MHDs) of at least moderate intensity for CM subjects, and monthly painkillers intake (MPI). Patients were also asked to rate pain severity (0–10 Numerical Rating Scale, NRS) of the monthly most painful attack and fill out migraine disability questionnaires (Headache Impact Test, HIT-6 [24], monthly, and the MIgraine Disability Assessing Scale [25], MIDAS, quarterly).

Patients were treated with galcanezumab subcutaneous injection with the first loading dose of 240 mg and then every month with 120 mg as recommended (www.europa.ema.eu). The above-reported variables and any adverse event (AE) were recorded at baseline and monthly at every in-office visit (from V1 to V6). Telephone/email contacts were allowed when in-office visits were not possible (e.g. isolation/quarantine due to Sars-Cov-2 pandemic). All AEs were reported to Eudravigilance and classified as gastrointestinal (e.g. nausea, constipation), cutaneous (e.g. injection-site reactions: rash/erythema, pruritus, urticaria, oedema/induration), arthralgia, Raynaud phenomenon, dizziness and other (< 1% of patients: i.e. somnolence, alopecia, anxiety).

The primary endpoint was to observe the change in MMDs (in HFEM patients) and MHDs (in CM patients) at the end of the sixth month of therapy compared to baseline. Secondary endpoints included changes in MPI, in NRS and in HIT-6 score and quarterly changes in MIDAS score, at V3) and V6 compared to baseline. Moreover, 50%, 75% and 100% responder rates (RR) were calculated for HFEM and CM groups at V1, V3 and V6. We also observed the prevalence of AEs.

All patients provided written informed consent. The study was approved by the Campus Bio-Medico University Ethical Committee n.30/20, mutually recognized by the other local ethical committees, and registered at the Italian Medicines Agency (Agenzia Italiana del Farmaco, AIFA) and at ClinicalTrials.gov NCT04803513.

Anonymized data will be shared by request from any qualified investigator.

### Statistical analysis

**Fig. 1:**
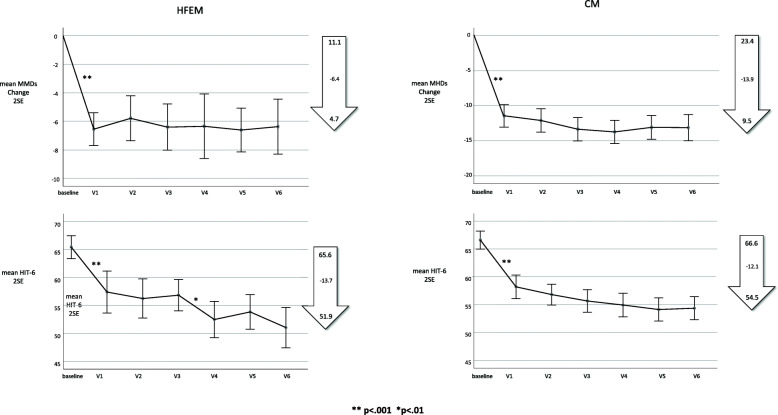
The left panel shows MMDs and HIT-6 score variations from baseline to V6 in HFEM group. The right panel shows MHDs and HIT-6 score variations from baseline to V6 in CM patients. ** *p* < .001

After the assessment of a preliminary cohort [19], we doubled the sample size. Statistical analyses were performed with SPSS version 26.0 (SPSS Inc., Chicago, IL, USA). As a priori analysis, non-parametric tests and contingency table (Chi-square and two-tailed Fisher exact tests) and unadjusted odds ratios (OR) with their 95% confidence intervals (CI) were run to compare variables between HFEM/CM or responder/non-responder patients. Interval variables were compared between groups with t-test (expressed as means with SD) or Mann-Whitney tests (medians with interquartile range [IQR]) according to the results of the Kolmogorov-Smirnov test for data distribution. Friedman analysis of rank was adopted to analyze the variable changes over time. However, since RCTs graphically reported the trend along times of MMDs/MHDs as means with standard error, to allow a graphic comparison with RCTs, Figs. 1 and 3 represent the considered variables as means. All tests were bilateral. Statistical significance was set as two-tailed *p* < 0.05. We initially investigated which clinical baseline characteristics associated with MMDs/MHD50% RR. After that, forced entry binary logistic regression investigated, which, among those resulting significantly related, confirmed the association to the responder condition. We included only subjects with complete information regarding the primary studied variables (MMDs and MHDs). For the secondary (HIT-6, MIDAS, NRS) variables, we declared data availability and ran the analysis only in patients with usable data.

## Results

To date, 245 patients have been enrolled in the GARLIT study. Of these 165 patients completed 6 months of observation since the first galcanezumab injections. For the current analysis, two subjects were excluded since the complete data set regarding primary studied variables were not available. We finally enrolled 163 patients (80.5% female, aged 47.1 ± 11.7 yrs., min-max 18–80 yrs). Of these, 10 patients (6.1%) dropped out for lack of effectiveness at least after 3 months of therapy, were included in the analysis as non-responders, and considered for the other endpoints for the treatment period.

At baseline, 130 (79.8%) patients were affected by CM, 33 (20.2%) by HFEM, 117 subjects (71.8%) also presented MO. Table 1 summarizes baseline demographical and clinical profiles in CM and HFEM patients. Among CM patients, 59 (45.4%) patients had previously failed OnabotulinumtoxinA treatment.

**Table 1:** Baseline demographic and clinical profiles in HFEM and CM patients

	HFEM (***n*** = 33)	CM (***n*** = 130)	***p***
**Age** (years. Mean. SD)	44.2 (11.7)	47.9 (16.7)	.127
**Sex** (%. Females)	82.4	80.6	1.000
**BMI** (kg/m^2^. median. IQr)	23.20 (4.25)	23.00 (3.68)	.487
**Comorbidities** (%)			
*Psychiatric*	18.8	20.5	1.000
*Gastrointestinal*	7.1	14.8	.367
*Vascular*	0	6.1	.345
*Hormonal*	14.3	10.5	.521
*Cancer*	10.7	1.8	.053
*Respiratory*	3.6	1.8	.485
*Diabetes*	0	2.7	1.000
*Hypertension*	7.1	15.8	.365
*Immuno- rheumatologic*	3.6	4.4	1.000
*Overweight*	36.4	28.6	.468
*Other*	17.9	12.1	.531
**MO** (%)	20.6	85.3	**<.001**
**Disease history** (years. Median. IQr)	26 (22)	30 (13)	.117
**Pain characteristics** (%)			.**033**
*throbbing*	46.9	70.7	
*dull*	50.0	28.5	
*other*	3.1	0.8	
**Dopaminergic features (%)**	57.1	64.0	.519
**Allodynia (%)**	43.8	67.5	**.023**
**Unilateral cranial autonomic features (%)**	46.4	48.2	.863
**Temporal artery hyperpulsatility (%)**	11.5	23.1	.274
**Number of failed preventives** (median. IQr. [min-max])	4 (3) [3–12]	5 (3) [3–12]	.**008**
**Triptan efficacy degree** (median. IQr)	2 (1)	1 (1)	**<.001**
**NRS (**median. IQr)	7 (1)	8 (1)	.**038**
**MMDs** (median. IQr)	11 (3)	20 (10)	**<.001**
**MHDs** (median. IQr)	11 (4)	21 (12)	**<.001**
**MPI** (median. IQr)	12 (5)	20 (15)	**<.001**
**HIT-6** (median. IQr)	66 (8)	68 (8)	.114
**MIDAS** (median. IQr)	30 (29)	72 (60)	**<.001**

*HFEM* high frequency episodic migraine, *CM* chronic migraine, *BMI* body mass index, *MO* medication overuse, *NRS* Numeric Rating Scale, *MMDs* monthly migraine days, *MHDs* monthly headache days, *MPI* monthly pain-killer intake, *HIT-6* headache impact test, *MIDAS* migraine disability assessment scale

The MMDs, MHDs and MPI were fully available during the evaluation times. From baseline to V6, HIT-6 and NRS score were regularly collected in 25 HFEM (73.5%) and 93 CM (72.1%) patients, and in 30 HFEM (90.9) and 108 CM (83.1%), respectively, while MIDAS in 20 HFEM (60.6%) and 77 CM (59.2%) patients.

### Episodic migraine

Patients reported a consistent decrease (*p* < .001) in MMDs from baseline 11 (IQr 3) to 4 (IQr 5) at V1, to 4 (IQr 5) at V3 and 3 (IQr 2) at V6; and in MPI from 12 (IQr 5) to 4 (IQr 6) at V1, to 4.5 (IQr 4) at V3 and 3 (IQr 2) at V6.

Disability presented from baseline a reduction (*p* < .001) in HIT-6 score from 66 (IQr 8) to 58 (IQr 8) at V1, to 55 (IQr 8) at V3, to 52 (IQr 11) at V6 and in MIDAS score from 30 (IQr 29) to 5 (IQr 10) at V3 and 3 (IQr 8) at V6.

Similarly, NRS reduced from baseline 7 (IQr 1) to 5 (IQr 2) at V1, to 6 (IQr 2) at V3, and to 5 (IQr 2) at V6, consistently (*p* < .001).

We observed a 50% MMD RR in 64.7% of patients at V1, 67.6% at V3 and 76.5% of cases at V6. The 75% RR was 32.4% at V1, 35.3% at V3 and 32.4% at V6. No patients achieved 100% MMD RR at V1, while it was observed in 5.9% of subjects at V3 and 11.8% at V6.

### Chronic migraine

Patients reported a consistent decrease (*p* < .001) in MMDs from baseline 21 (IQr 12) to 10 (IQr 12) at V1, to 9 (IQr 11) at V3 and 7 (IQr 10) at V6; and in MPI from 20 (IQr 15) to 7 (IQr 8) at V1, to 7 (IQr 6) at V3 and 5 (IQr 8) at V6.

Disability presented from baseline a reduction (*p* < .001) in HIT-6 score from 68 (IQr 34) to 61 (IQr 11) at V1 to 56 (IQr 15) at V3 to 55 (IQr 14) at V6, and in MIDAS score from 72 (IQr 60) to 22 (IQr 44) at V3 and to 18 (IQr 43) at V6.

Similarly, NRS reduced from baseline 8 (IQr 1) to 6 (IQr 2) at V1 to remain stable at V3 and V6 consistently (*p* < .001).

We observed a 50% MMD RR in 54.3% of patients at V1, 66.7% at V3 and 63.5% of cases at V6. The 75% RR was 22.5% at V1, 33.3% at V3 and 37.8% at V6. No patients achieved 100% MMD RR at V1, while it was observed in 2.3% of subjects at V3 and 7.1% at V6.

**Fig. 2:**
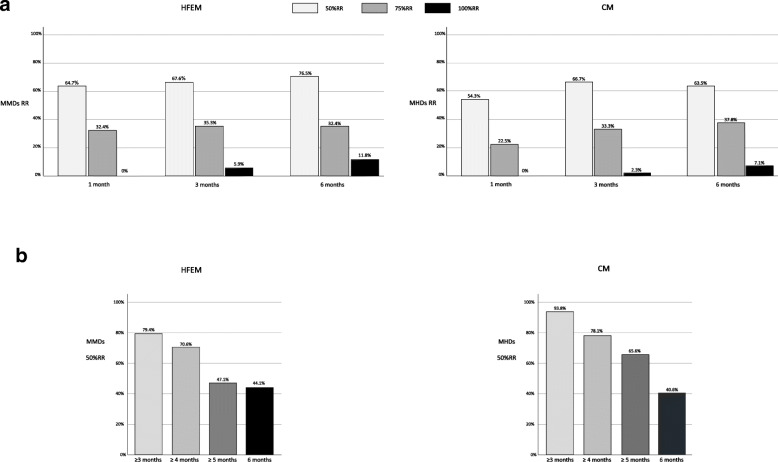
Panel **a** displays: 50%, 75%, 100% MMDs RR after 1, 3 and 6 months of therapy in HFEM patients (on the left) and 50%, 75%, 100% MHDs RR after 1, 3 and 6 months of therapy in CM patients (on the right). Panel **b** graphically shows the percentage of HFEM patients achieving at least 3, 4, 5 or 6 cumulative months with 50% MMDs RR (on the left) and the percentage of CM patients achieving at least 3, 4, 5 or 6 cumulative months with 50% MHDs RR (on the right)

Figure 1 shows MMDs (in HFEM patients), MHDs (in CM group), and HIT-6 score (for both CM and HFEM) from baseline to V6. The main effect was observed in the first month of therapy for all variables while from V2 to V6 only HIT-6 presented a further significant (*p* = .009) decrease from V3 (55 IQr 11) to V4 (52 IQr 3). Figure 2a summarizes RRs for MMDs and MHDs in HFEM and CM patients, respectively. Figure 2b shows the percentage of CM and HFEM achieving at least 3, 4, 5 or 6 cumulative months with MHDs and MMDs 50% RR, respectively.

**Fig. 3:**
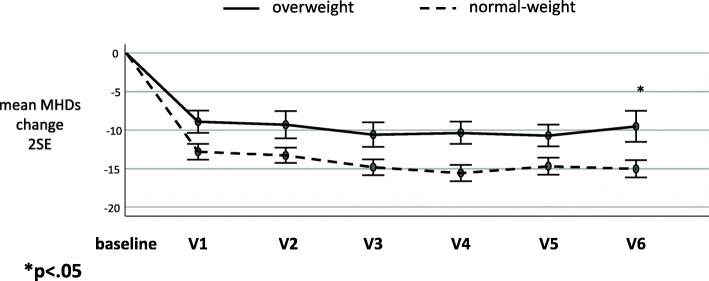
The graphic shows MHDs variations from baseline to V6 in normal-weight compared with over-weight CM patients

In CM patients, 6-month MHDs ≥50% responder patients presented a lower body mass index (24.20 IQr 4.05 vs 22.40 IQr 4.10; *p* = .018) and had failed fewer preventive treatments (4 IQr 2 vs 6 IQr 4; *p* = .013). Binary logistic regression confirmed these associations (Table 2). No other baseline characteristics in CM patients and none in the HFEM groups differentiated responders from non- responders. Figure 3 outlines MHDs variation over time in CM as compared for overweight, MHDs differed statistically at V6 (*p* = .025; − 14.00 IQr 12.75 vs − 15.00 IQr 13.75).

**Table 2:** Binary logistic regression analysis on 50%MHD Responder Rate in CM

	B	S.E.	Wald	Significance	Odds ratio	95% C.I.	
						Lower	Upper
Age	.026	.021	1.473	.225	1.026	.984	1.070
Sex	.155	.574	.073	.787	1.168	.379	3.599
**BMI**	**−.107**	**.051**	**4.429**	**.035**	**.899**	**.814**	**.993**
**Failed preventive treatments**	**−.266**	**.114**	**5.420**	**.020**	**.766**	**.612**	**.959**

*BMI* body mass index, *S.E* standard error

In EM patients, we found no association between clinical variables and 6-months MMD 50%RR.

Conversion from CM to EM was observed in 73.6% of patients at V3 and 77.2% of patients at V6. Patients no longer presented MO in 82.9% of cases at V3 and 82.0% at V6.

**Fig. 4:**
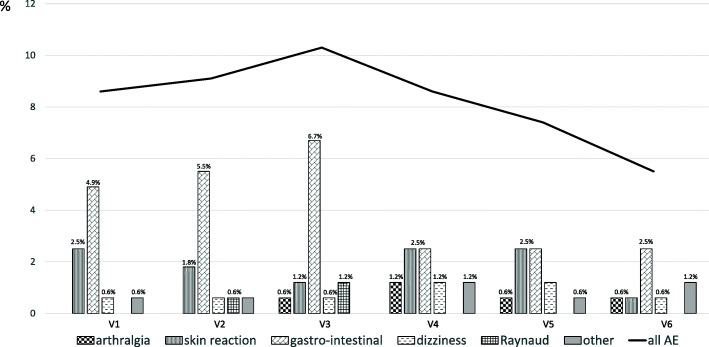
The frequency (%) of adverse events and their trend over evaluation times are detailed

Figure 4 details types of adverse events and their course over time.

Most common events were constipation and injection-site reactions (> 2% of patients). Other events, such as dizziness, arthralgia and Raynaud phenomenon, were present in < 2% of patients throughout the study. All the AEs were most common at the start of the treatment/in the first 3 months and tended to resolve in the following months.

Finally, as no AE induced no patient to cease the treatment, the discontinuation rate, i.e. the percentage of patients who interrupted the treatment, was due only to those patients who interrupted galcanezumab injections mainly for lack of effectiveness (6.1%) as reported above. In detail, 3 patients ceased treatment after 3 months of therapy, 3 after 4 months of therapy and the remaining 4 after 5 months of therapy. Only one of these patients dropped out after 5 months of therapy for coexistence of urticarial and inefficacy.

## Discussion

Our 6-month study demonstrated that galcanezumab appears effective, safe, and well tolerated to prevent migraine in CM and HFEM patients also in real life. We found that the MHD and MMD reductions were more extensive than those observed in the three RCTs. MMDs variation (i.e., − 8 days) was almost double in the EM group compared to the EVOLVE studies (i.e. -4.7 and − 4.3), and more than twice (i.e. -13) in the CM group versus the REGAIN (− 4.8) at 3 months. The reduction in terms of migraine headache days occurred mainly after the first-month loading dose, followed by a slightly further decrease in the following months till V6. This rapid response characterizes the use of galcanezumab, as also recently described in the post-hoc analysis of the CONQUER [26].

Our study enrolled hard-to-treat migraine patients who had failed at least three preventives. These patients, poorly represented in EVOLVE and REGAIN trials, were more frequently enrolled in the CONQUER study [27], which included patients with previous failure of 2 to 4 preventives. It observed a reduction of 6 MHDs in CM patients at 3 months [27] and 8.6 MHDs in the overall population at 6 months [28]. In both evaluation times, the benefit observed in our study was much larger.

Similarly, disability improved more substantially than in RCTs. In particular, compared to the REGAIN study, our CM patients reported a higher MIDAS score at baseline (72 vs 62 points) but perceived an impressive reduction of 50 points (vs 22) after 6 months of treatment. Accordingly, we noted a progressive reduction also in HIT-6 score from baseline to month 6 in both EM and CM groups (Fig. 1).

The proportion of patients with a 50% reduction in MHDs compared to baseline is probably considered worldwide as the adequate cut-off point to define the efficacy of prophylactic treatments. The EVOLVE trials reported a 50%RR at 6 months in about 60% of EM patients, while the 50%RR observed in the REGAIN study in CM patients at 3 months was 27.6%. In our EM group, the MMD 50%RR was 76.5% at 6 months, while among CM patients, the MHD 50%RR was 66.7% at 3 months. Moreover, it is worth noting that 11.8% of EM and 7.1% of CM patients had no headaches (i.e. 100% RR) after the sixth month of treatment.

Persistence is another point of paramount importance to qualify the efficacy of a treatment. Among RCTs galcanezumab-treated EM patients, the sustained MHD 50%RR over 6 months was about 20% and about 41% over ≥3 months; in CM patients, the consecutive MHD 50%RR over 3 months was about 17% [29]. In the GARLIT study, 44% of EM and 40% of CM subjects consistently presented a 50%RR during the entire 6 months of therapy, and around 80% of EM and over 90% of CM patients achieved 50%RR for at least 3 months.

In our analysis, galcanezumab responsiveness seemed positively associated with a lower BMI and fewer failed preventives at baseline.

Epidemiological studies have suggested obesity as a risk factor for chronic migraine, although a clear causal relationship has not been established [30]. However, evidence supports the role of CGRP as a potential molecular link between obesity and migraine: a) inflammatory markers are elevated both in obesity and migraine, b) adipose tissue secretes pro-inflammatory cytokines and adipocytokines, implicated in migraine pathophysiology and c) elevated plasma levels of CGRP were detected in obese individuals [31]. Thus, enhanced trigeminal CGRP production in obese individuals may lower the threshold to trigger migraine attacks, leading to more frequent episodes (and eventually to chronic migraine). In this scenario, overweight patients might require a more aggressive CGRP pathway inhibition or a multi-targeted approach.

The positive association between 50%RR and a lower number of preventive medication failures, also observed in erenumab real-life studies [17], is somewhat intuitive but not commonly described in the headache literature. On the other hand, this issue has been widely discussed in epilepsy, a disease often compared with migraine for possible common pathophysiologic mechanisms and the high percentage of treatment failures. Prior exposure and lack of response to commonly used antiepileptic drugs (AEDs) predict the failure to a new AED in patients with drug-resistant focal epilepsy [32]. The more prior failures, the less the probability to benefit from the new one. Likely, the drug mechanism of action does not play a significant role in this phenomenon, as also switching to new therapeutic targets produced similar results. Various factors may contribute to the reduced efficacy observed in different patient populations; genetic variations may explain most inter-individual variability in response to AEDs among patients [33]. Other mechanisms should also be considered in migraine patients, such as psychiatric and other comorbidities or aberrant cerebral plastic phenomena, which can be very difficult to revert [34].

In the GARLIT study galcanezumab was well tolerated, and no serious AEs emerged. The most common AEs reported were constipation and injection-site reactions (2%). These events occurred mainly in the first months and then tended to resolve. As no AE induced no patient to cease the treatment, the discontinuation rate was due only to those patients who interrupted galcanezumab injections for lack of effectiveness (6.1%). Besides, no patients experienced cardio- and cerebrovascular events, confirming the vascular safety of blocking the CGRP pathway described in clinical trials and experimental studies [8, 35]. The point is of pivotal importance as discontinuation rate makes another substantial difference with the oral preventive therapies, having a very low adherence (about 25% at 6 months and 14% at 12 months [7]). Our study confirms this issue in real life, even in hard-to-treat patients with previous several failed migraine preventives.

The RCTs provide evidence of the highest grade, whereas observational studies are believed to overestimate treatment effects [36]. However, well-designed real-world observational studies usually provide reliable information and should be used to share clinical experience among experts and ameliorate everyday clinical practice [37]. Patients enrolled in most galcanezumab RCTs do not adequately reflect the population we can treat in the real world, at least in Italy, where mAbs are reimbursed only for HFEM and CM patients with three or more failed preventives and moderate-severe disability. Our patients were representative of migraineurs seen in everyday clinical practice and were quite similar to the CONQUER cohort. Accordingly, the discrepancies between the results of GARLIT and CONQUER studies are less relevant than those observed with the other galcanezumab RCTs.

Most patients participating in our study had been followed by the participating centers for a long time before the study enrollment, allowing an accurate diagnosis and definition of previous treatment responses and probably a better selection of patients. In our opinion, a more accurate selection and the consecutive enrollment of patients considered eligible for treatment based on the rules enacted by Italian authorities may make the difference, as observed for previous real-life studies [17]. Alternatively, our results could be highly affected by a placebo effect. Longer follow-up will help clarify this aspect, as the placebo effect usually decreases over time [38].

## Conclusions

While RCTs are the milestone to establish new therapies’ efficacy, real-life studies are necessary to

optimize the use of a new treatment in more complex clinical settings. The benefit of galcanezumab in preventing migraine attacks in HFEM and CM patients was more remarkable in the GARLIT real-life study than in RCTs. Galcanezumab proved to alleviate patients rapidly also in real-life and to offer sustained benefit during the entire treatment period in a fair proportion of subjects. On the other hand, some conditions such as a higher BMI and a history of multiple preventive therapy failures can characterize harder-to-treat patients. Further studies with longer follow-up and wider samples are necessary to demonstrate other possible useful indicators and the persistence of efficacy and tolerability of galcanezumab in the real world.

## Data Availability

Anonymized data will be shared by request from any qualified investigator.

## Abbreviations

AE: Adverse event
AED: Antiepileptic drug
AIFA: Agenzia Italiana del Farmaco (Italian Medicines Agency)
BMI: Body mass index
CI: Confidence intervals
CGRP: Calcitonin gene-related peptide
CM: Chronic migraine
EM: Episodic migraine
HFEM: High frequency episodic migraine
HIT-6: Headache Impact Test 6 items
IQr: Interquartile ranges
MHDs: Monthly Headache Days
MMDs: Monthly Migraine Days
mAb: Monoclonal antibody
MPI: Monthly Painkillers Intake
NRS: Numerical Rating Scale
MIDAS: Migraine Disability Assessment
RCT: Randomized controlled trials
RR: Responder rate
